# Dissemination and genetic diversity of chlamydial agents in Polish wildfowl: Isolation and molecular characterisation of avian *Chlamydia abortus* strains

**DOI:** 10.1371/journal.pone.0174599

**Published:** 2017-03-28

**Authors:** Monika Szymańska-Czerwińska, Agata Mitura, Krzysztof Niemczuk, Kinga Zaręba, Agnieszka Jodełko, Aneta Pluta, Sabine Scharf, Bailey Vitek, Rachid Aaziz, Fabien Vorimore, Karine Laroucau, Christiane Schnee

**Affiliations:** 1 Department of Cattle and Sheep Diseases, National Veterinary Research Institute, Pulawy, Poland; 2 Department of Biochemistry, National Veterinary Research Institute, Pulawy, Poland; 3 Institute of Molecular Pathogenesis, Friedrich-Loeffler-Institut (Federal Research Institute for Animal Health), Jena, Germany; 4 University Paris-Est, Anses, Animal Health Laboratory, Bacterial Zoonoses Unit, Maisons-Alfort, France; University of Technology Sydney, AUSTRALIA

## Abstract

Wild birds are considered as a reservoir for avian chlamydiosis posing a potential infectious threat to domestic poultry and humans. Analysis of 894 cloacal or fecal swabs from free-living birds in Poland revealed an overall *Chlamydiaceae* prevalence of 14.8% (n = 132) with the highest prevalence noted in Anatidae (19.7%) and Corvidae (13.4%). Further testing conducted with species-specific real-time PCR showed that 65 samples (49.2%) were positive for *C*. *psittaci* whereas only one was positive for *C*. *avium*. To classify the non-identified chlamydial agents and to genotype the *C*. *psittaci* and *C*. *avium*-positive samples, specimens were subjected to *omp*A-PCR and sequencing (n = 83). The *omp*A-based NJ dendrogram revealed that only 23 out of 83 sequences were assigned to *C*. *psittaci*, in particular to four clades representing the previously described *C*. *psittaci* genotypes B, C, Mat116 and 1V. Whereas the 59 remaining sequences were assigned to two new clades named G1 and G2, each one including sequences recently obtained from chlamydiae detected in Swedish wetland birds. G1 (18 samples from Anatidae and Rallidae) grouped closely together with genotype 1V and in relative proximity to several *C*. *abortus* isolates, and G2 (41 samples from Anatidae and Corvidae) grouped closely to *C*. *psittaci* strains of the classical ABE cluster, Matt116 and M56. Finally, deep molecular analysis of four representative isolates of genotypes 1V, G1 and G2 based on 16S rRNA, IGS and partial 23S rRNA sequences as well as MLST clearly classify these isolates within the *C*. *abortus* species. Consequently, we propose an expansion of the *C*. *abortus* species to include not only the classical isolates of mammalian origin, but also avian isolates so far referred to as atypical *C*. *psittaci* or *C*. *psittaci/C*. *abortus* intermediates.

## Introduction

The family *Chlamydiaceae* comprises a group of obligatory intracellular bacteria within the single genus *Chlamydia* (*C*.) which includes eleven species [1] and two candidate species [2–3]. Chlamydiae are widely distributed throughout the world, causing a variety of diseases both in humans and animals, including zoonotic infections. Avian chlamydiosis due to *Chlamydia psittaci* was reported for the first time at the end of the 19^th^ century and became of world concern in 1930 after the large epidemic involving psittacine birds and affecting 750–800 individuals in America and Europe [4]. In birds, the disease is characterized by respiratory, ocular and enteric symptoms occasionally with fatal outcome, but asymptomatic, latent infections are also common. Shedding of the pathogens through feces or ocular and respiratory secretions occurs intermittently in both diseased birds and asymptomatic carriers, thus representing a reservoir of infection for birds and humans [5]. Based on the *omp*A gene which encodes the major immunogenic protein of chlamydiae, avian *C*. *psittaci* has been classified into fifteen genotypes, each one more or less closely associated with certain bird species. Seven of these genotypes (A-F, E/B) are predominant whereas the other eight genotypes (1V, 6N, Mat116, R54, YP84, CPX0308, I and J) were described as provisional [6–10]. Whereas *C*. *psittaci* had been considered for a long time to be the sole species hosted by birds, recent evidence suggested that other chlamydial species, such as *C*. *abortus*, *C*. *pecorum*, *C*. *trachomatis*, *C*. *suis* and *C*. *muridarum* [11–13], can also be harboured by birds as well as the recently described avian species *C*. *gallinacea* and *C*. *avium* [14].

Wild birds with chlamydiosis draw attention when outbreaks with die-offs are noted [15], nevertheless, latent and asymptomatic infections seem to be the rule. Representatives of Psittaciformes and Columbiformes are the most prominent hosts for chlamydiae [5], but prevalence studies revealed their occurrence, for example, also in Anseriformes, Charadriiformes, Passeriformes, Falconiformes, Accipitriformes or Procellariformes [8, 16–20]. A recent survey in wildfowl from Poland focusing on wetland birds [21] found an overall prevalence of 7.4% with *C*. *psittaci* as the predominant chlamydial agent in cormorants and mallards. Interestingly, many studies report the detection of atypical or non-typable *Chlamydiaceae* in wild birds [8–9, 16, 21], however, their further characterization was often hampered by unsuccessful cultivation attempts and, thus, the lack of isolates from the unknown species or genotypes. Therefore, the aim of our study was not solely the collection of prevalence data for *Chlamydiaceae* in wild birds in ten out of sixteen Polish districts (voivodeships), but also the isolation of chlamydial agents to enable their in-depth molecular characterization as well as further investigations into epidemiology, host preference, pathogenicity and zoonotic potential.

## Materials and methods

### Samples

**Fig 1:**
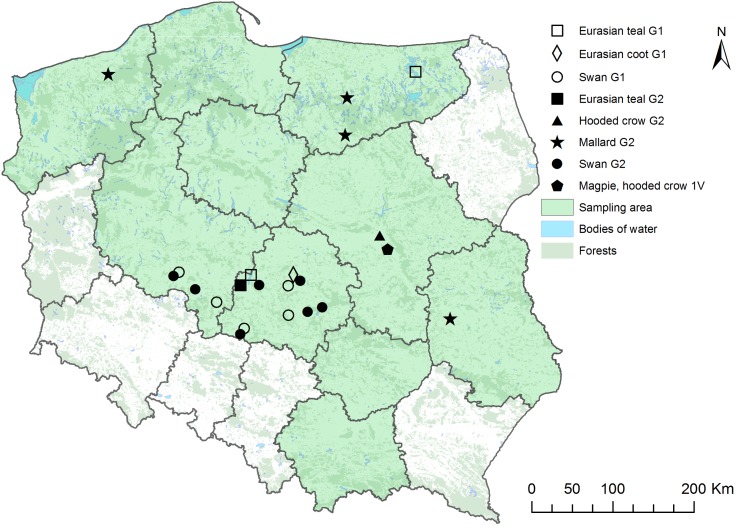
Areas of bird sampling in Poland (ArcMap 10.4 software).

Cloacal or fecal swabs (n = 894) were collected from different species of feral birds belonging to 16 families (**Table 1**). Samples were collected from birds transiently living in bird rehabilitation centres and from free-living birds caught randomly by authorised veterinarians or ornithologists during clinical studies or routine activities following standard procedures. The individual health status of a bird was not recorded. Sampling was performed between July 2014 and October 2015 in different regions of Poland (**Fig 1**). For DNA extraction, dry swabs were stored at −20°C, and for chlamydia isolation, swabs were placed in Chlamydia stabilizing medium or PBS buffer and stored at -80°C.

**Table 1:** Results summary of free-living birds testing.

			Real-time PCR		*omp*A sequencing							Final clasiffication			
					Seq no	*C*. *psittaci*			avian *C*. *abortus*						
Family	Bird species	Birds no	*Chlamydiaceae*	*C*. *psittaci*		B	C	Mat116	G1	G2	1V	*C*. *avium*	*C*. *psittaci*	avian *C*.*abortus*	Non-classified *Chlamydia*
Accipitridae*	common buzzard	5	0	0											
	western marsh harrier	2	0	0											
	Eurasian sparrowhawk	1	0	0											
	**Subtotal**	**8**	**0**	**0**											
**Anatidae**	mallard	301	23 (7.6%)	8 (34.8%)	11		4			6		1	6	6	10
	swan^1^	210	78 (37.1%)	49 (62.8%)	55		8		14	33			16	47	15
	Eurasian teal	18	3 (16.7%)	1 (33.3%)	3				2	1				3	
	greylag goose	3	0	0											
	bean goose	1	0	0											
	garganey	1	1 (100%)	0											1
	**Subtotal**	**534**	**105 (19.7%)**	**58 (55.2%)**	**69**		**12**		**16**	**40**		**1**	**22**	**56**	**26**
**Apodidae**	common swift	29	1 (3.5%)	0											1
Ardeidae*	heron	2	0	0											
	little bittern	1	0	0											
	**Subtotal**	**3**	**0**	**0**											
**Ciconiidae**	white stork	38	1 (2.6%)	0											1
**Columbidae**	pigeon^2^	64	3 (4.7%)	1 (33.3%)	1	1							1		2
**Corvidae**	magpie	36	12 (33.3%)		6						6			6	6
	hooded crow	52	4 (7.7%)	2 (50%)	3	1				1	1		2	2	
	jack daw	28	1 (3.6%)	0											1
	Eurasian jay	8	0	0											
	common raven	3	0	0											
	**Subtotal**	**127**	**17 (13.4%)**	**2 (11.8%)**	**9**	**1**				**1**	**7**		**2**	**8**	**7**
Falconidae*	common kestrel	2	1 (50.0%)	0											1
Hirundinidae*	common house martin	4	0	0											
	swallow	2	0	0											
	**Subtotal**	**6**	**0**	**0**											
Laniidae*	red-backed shrike	1	0	0											
**Laridae**	gull^3^	63	2 (3.2%)	2	2			2					2		
Paridae*	great tit	1	0	0											
Prunellidae*	dunnock	1	0	0											
Rallidae*	Eurasian coot	2	2	2	2				2					2	
Strigidae*	tawny owl	2	0	0											
	long-eared owl	2	0	0											
	Eurasian eagle-owl	1	0	0											
	**Subtotal**	**5**	**0**	**0**											
Turdidae*	song thrush	5	0	0											
	common blackbird	3	0	0											
	fieldfare	2	0	0											
	**Subtotal**	**10**	**0**	**0**											
**TOTAL**		**894**	**132 (14.8%)**	**65 (49.2%)**	**83**	**2**	**12**	**2**	**18**	**41**	**7**	**1**	**27**	**66**	**38**

* If the number of samples in each tested group was less than 10, the group has been excluded from statistical analysis.

^1^ Swan: mute swan (n = 208), whooper swan (n = 2).

^2^ Pigeon: feral pigeon (n = 58), Common wood pigeon (n = 4), Eurasian collared dove (n = 2).

^3^ Gull: black-headed gull (n = 46), European herring gull (n = 12), common gull (n = 5).

### Ethics statement

According to the Local Ethical Committee on Animal Testing at the University of Life Sciences in Lublin (Poland), formal ethical approval is not required for this kind of study. Guidelines published by this ethics committee [22] were consulted, which confirm that this work is sanctioned without specific ethical approval. Permissions for sampling from authorities of Rehabilitation Centre of Protected Birds in Warsaw and Rehabilitation Centre of Wild Birds localised in Department of Animal Surgery, University of Environmental and Life Sciences in Lublin were obtained. Activity of rehabilitation centres for birds is regulated by Polish law—Act of 16 April 2004 on nature conservation (Polish Journal of Laws 2004, No 92 item 880) [23]. All samples, including these from protected species, were taken by veterinarians during routine medical and veterinary activities e.g. health status controls or surgery. Part of samples were taken by ornithologists during Multiannual Research Programme, Free-living water birds as a reservoirs and vector in the spread avian influenza” conducted at Department of Poultry Diseases, National Veterinary Research Institute in Pulawy, Poland or during birds ringing based on Regulation of the Minister of the Environment of 14 March 2006 on bird ringing [24]. Ministry of the Environment issued appropriate permissions for this type of activity on the territory of Poland for ornithologists and workers of rehabilitation centers.

### DNA extraction

QIAamp DNA Mini Kit (Qiagen, Germany) was used for the DNA extraction from cloacal and fecal swabs according to the manufacturer's instructions but with one modification: DNA extracts were eluted in 100 μL instead of 200 μL of elution buffer (AE). DNA extracts were stored at −20°C before analysis.

### Real-time PCR

All DNA extracts (n = 894) were screened using a *Chlamydiaceae-*specific real-time PCR targeting the 23S rRNA gene fragment, as previously described in Ehricht *et al*. [25] In order to distinguish true target negatives from PCR inhibition, the internal positive control (TaqMan Exogenous Internal Positive Control, Applied Biosystems) was added to the reaction mixture. An analytical cut-off value of 39 was selected corresponding to the defined lower limit of detection of the test. Any Ct value above this defined limit would, thereafter, be considered not reliable.

All *Chlamydiaceae*-positive samples were re-tested with the *inc*A-based *C*. *psittaci* real-time PCR [26] and with the 16S rRNA-based *C*. *avium* real-time PCR [27]. Selected samples were also tested with a *C*. *abortus*-specific real-time PCR targeting the *omp*A gene [11].

### Sequencing of *omp*A and *rrn* genes

Amplification of the partial *omp*A gene (approximately 1050 bp and 1200 bp) was based on protocols by Madani *et al*. [9] and Sachse *et al*. [6], respectively. Primer sets CTU 5′-ATGAAAAAACTCTTGAAATCGG-3′ / CTL 5′-CAAGATTTTCTAGA(T/C)TTCAT(C/T)TTG-3′ and CTU / *omp*A-rev (5'-TCC TTA GAA TCT GAA TTG AGC-3') were used. PCR products were sent to companies (Genomed, Poland and Eurofins Genomics, Germany) for sequencing. Sequences of the partial 16S rRNA gene, intergenic spacer and partial 23S rRNA gene were amplified in two separate PCRs according to protocols by Everett et al.[28], Pudjiatkomo et al. [29] and Thomas et al. [30] using primer sets 16S1 5’-CGGATCCTGAGAATTTGATC-3’ / rp2 5’- CTACCTTGTTACGACTTCAT-3’ and 16SF2 5’-CCGCCCGTCACATCATGG-3’/ 23SIGR 5’-TGG CTC ATC ATG CAA AAG GCA-3’.

The partial *omp*A and *rrn* sequences from the present study were submitted to GenBank (NCBI) and registered under accession numbers KX062046–KX062087, KX424651–KX424673, KX 870482-KX870499 and KX870500-KX070503 (**S1 Table).**

### Sequence comparison and phylogenetic analysis

PCR products of the *omp*A gene obtained from 83 Chlamydia-positive samples were sequenced and the data analysed using Geneious Pro 8.0 software (Biomatters, New Zealand). Sequences were subjected to BLAST analysis against the GenBank database (NCBI) to identify related sequences and, finally, aligned with a panel of sequences representing *C*. *felis*, *C*. *caviae*, *C*. *abortus*, as well as *C*. *psittaci* including the majority of the currently described genotypes: A, B, E/B, C, D, E, F, I, Mat116, M56, WC, YP84, and 1V, as well as four sequences previously detected in Swedish wetland birds, namely 60194, 1192, 50338 and 55435. For dendrogram construction, sequences were trimmed to 850 bp.

Sequences obtained from chlamydial isolates in two different PCR amplifications targeting the *rrn* locus were concatenated in order to increase the resolution of the phylogenetic analysis. They were aligned with the most related and relevant sequences from the GenBank database (NCBI) including *rrn* sequences from a wide variety of *C*. *abortus* and *C*. *psittaci* strains and trimmed to fragments of 2305 bp.

A DNA substitution Hasegawa-Kishono-Yano (HKY85) model with the best fit for analysis of the *C*. *psittaci omp*A and *rrn* sequences was calculated using Datamonkey webserver [31]. Dendrograms were constructed using Neighbor Joining (NJ) with the robustness of the clusters assessed by bootstrapping 1000 replicates. Mean nucleotide distances among *C*. *psittaci/C*. *abortus* genotypes were estimated by adopting the same model in MEGA 6 software [32].

### MLST analysis

MLST analysis was performed based on previous publications [33–34]. Multiple alignments of the seven concatenated MLST gene fragments were conducted using Geneious Pro 8.0 software and a dendrogram was constructed using NJ. For dendrogram construction, sequences were trimmed to 3104 bp. MLST sequences from the present study have been deposited at GenBank (NCBI) under accession numbers KX870454-KX870481.

### Isolation and propagation in cell culture

Buffalo green monkey (BGM) cells in minimal essential medium (MEM, Lonza, Cologne, Germany) with 5% serum were seeded into Trac bottles containing glass coverslips (Bibby Sterilin Ltd., Staffordshire, UK) and incubated at 37°C with 5% CO_2_ in a fully humidified cabinet for 4 days. Swabs in 1–2 mL Chlamydia stabilizing medium or PBS buffer were ultrasonicated (ten 0.8-s pulses with 0.2-s pause between each pulse at an amplitude of 80%; Branson 450D Sonifyer) and 30–300 μL of medium or buffer were inoculated into six Trac bottles with confluent grown BGM monolayers. After inoculation, the bottles were centrifuged at 3000 × *g* and 37° C for 60 min and subsequently incubated for 2 h. The MEM medium was then replaced with serum-free medium UltraMDCK (Lonza Cologne, Germany) containing the antibiotics amphotericin (2.5 μg/mL), gentamicin (10 μg/mL) and vancomycin (25 μg/ mL). The medium was renewed after 18 h. Three days after inoculation, a single coverslip was fixed with methanol, and the monolayer was stained with IMAGEN Chlamydia (Oxoid Ltd., Cambridgeshire, UK). A sample was considered positive when inclusions of typical chlamydial morphology appeared as bright apple-green spots after two passages.

### Statistical analysis

All analyses were conducted using the program STATISTICA ver. 10 (StatSoft, part of Dell Software, USA). Correlation among dependent variables of interest such as prevalence of *Chlamydiaceae* shedders among bird families and species was calculated. Testing was carried out using the chi-squared test with appropriate Bonferroni correction. In order to evaluate the level of *Chlamydiaceae* shedding in bird family and species the Kruskal–Wallis nonparametric test was carried out. The odds ratios (OR) were also calculated in order to assess the chance of shedding of *Chlamydiaceae* in bird families and individual species within families.

## Results

### *Chlamydiaceae* detection

Results of the *Chlamydiaceae*-specific real-time PCR for different bird families are presented in **Tables 1** and **S1**. Among 894 specimens, 132 were positive, giving a prevalence value of 14.8%. Anatidae and Corvidae families demonstrated the highest level of *Chlamydiaceae* prevalence at 19.7% (Ct values ranging from 19.1 to 38.4, mean Ct 31.7) and 13.4% (Ct values ranging from 30.8 to 38.5, mean Ct 36.2), respectively. The highest percentages of *Chlamydiaceae*-positive cloacal/fecal swabs were obtained in two bird species: mute swan (37.1%), and magpie (33.3%). Mean Ct values of 34.4 (Ct range from 22.4 to 38.45) and 30.9 (Ct range from 19.1 to 38.0) were observed for mallards and swans, respectively. Positive Eurasian teal samples (16.7%) showed a mean Ct value of 33.3 (ranging from 27.5 to 36.6), whereas a single garganey was positive with Ct 36.0. The percentage of positive samples in Columbidae family was 4.7% with a Ct range from 34.4 to 36.3 (Ct mean value 35.2). Laridae family members demonstrated a 3.2% level of prevalence of *Chlamydiaceae* with two positive samples (Ct mean values 34.4, ranging from 31.5 to 37.4). In Apoidae and Ciconiidae families, 3.5% and 2.6% of specimens, respectively, were shown to harbour chlamydiae. The presence of chlamydial DNA was also confirmed in one out of two samples from kestrels belonging to the Falconidae family (Ct 35.7), as well as in Eurasian coot (n = 2) of the Rallidae family (Ct mean value 35.5, ranging from 35.0 to 36.0). *Chlamydiaceae* were not detected in birds belonging to the Ardeidae, Accipitridae, Hirundinidae, Laniidae, Paridae, Prunellidae, Strigidae, or Turdidae families.

### Statistical analysis of *Chlamydiacae* prevalence and shedding levels

The chi-squared test confirmed statistically significant differences of *Chlamydiaecae* prevalence between families only for Anatidae and Laridae (p = 0.001), but it confirmed significant differences between species within families in several cases: in Anatidae, between mallards and swans (p<0.0001); and in Corvidae, between magpies and hooded crows (p = 0.002) as well as between magpies and jackdaws (p = 0.003). The odds ratio (OR = 7.5) showed that the probability of occurrence of *Chlamydiaceae* in Anatidae is 7.5 times higher than in Laridae. Moreover, the probability of *Chlamydiaceae* shedding is more than 7 times higher in swans than in mallards. The Kruskal-Wallis test confirmed statistically significant differences between bird families in terms of shedding levels reflected by average Ct values (p = 0.022). The post-hoc test showed that a significant difference was noted only between the families Anatidae and Corvidae (p = 0.006). Interestingly, a substantial variation of shedding levels was noted for mallards and swans (p = 0.025).

### Species identification by species-specific real time PCR and *omp*A analysis

Further testing was conducted with species-specific real-time PCR (results shown in **Tables 1** and **S1**). In total, 65 samples (49.2%) were positive with the *C*. *psittaci-*specific real-time PCR and only one with the *C*. *avium*-specific real-time PCR, whereas no positives were obtained with the *C*. *abortus*-specific PCR assay. In order to classify the remaining so far unknown chlamydial agents and to genotype the *C*. *psittaci* and *C*. *avium*-positive samples, specimen with Ct values below 37 in the *Chlamydiaceae*-PCR were subjected to *omp*A-PCR and sequencing. *Omp*A sequences were successfully obtained for 83 samples.

**Fig 2:**
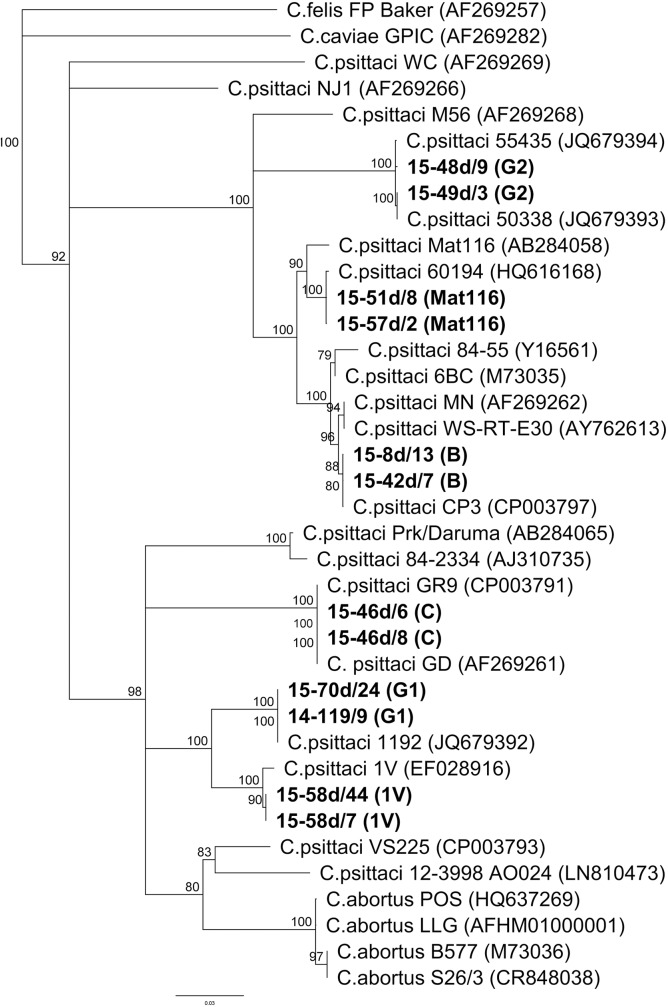
*omp*A-based NJ dendrogram constructed from a global alignment of 850 bp. Specimens found in this study are in boldface. Representative sequences from various *C*. *psittaci* genotypes and *C*. *abortus* are included. The tree was rooted on the sequence of *C*. *felis* (FP Baker).

A NJ dendrogram was constructed using an 850 bp alignment of *omp*A sequences from representative strains of *C*. *psittaci*, *C*. *abortus*, *C*. *caviae* and C. *felis* available from GenBank. Compared to those sequences, the sequences from our study grouped into six clades. In **Fig 2**, the *omp*A dendrogram is shown with two representative sequences for each of these six clades. Indeed, 23 out of 83 sequences were assigned to four clades representing the previously described *C*. *psittaci* genotypes B, C, Mat116 and 1V. Sequences obtained from a pigeon (15-42d/7) and a hooded crow (15-8d/13) grouped to genotype B represented by *C*. *psittaci* CP3 (CP003797), with a bootstrap value of 82.3% and 100% pairwise identity. Twelve sequences isolated from four mallards (15-50d/1,15-53d/16, 15-64d/1, 14-99d/1) and eight swans (15-46d/6, 15-46d/8, 15-46d/10, 15-49d/6, 15-53d/8, 15-64d/1, 15-78d/8, 15-78d/10) were classified as genotype C represented by type strain GR9 (CP003791) with a maximum 100% bootstrap support. The sequences from these twelve specimens and from the type strain GR9 were identical. Two sequences isolated from gulls (15-57d/2 and 15-51d/8) were assigned to genotype Mat116, together with sequences previously isolated from a white-tailed sea-eagle and a budgerigar, with 100% bootstrap support. The highest degree of similarity (99.9%) was found to the sequence from a white-tailed sea-eagle, whereas similarity with Mat116 from a mini-macaw was only 98.2%. Seven sequences isolated from a hooded crow (15-58d/28) and six European magpies (15-58d/19, 15-58d/7, 15-58d/11, 15-58d/31, 15-58d/43, 15-58d/44) were classified as genotype 1V represented by a hooded crow sequence from Russia, with a bootstrap value of 100%. They differed in five to six point mutations from the Russian crow sequence and were 99.9% similar to each other with only a single nucleotide substitution.

The remaining 59 sequences formed two new clades together with recently described sequences from chlamydiae in Swedish wetland birds [16]. Thus, eighteen sequences isolated from two Eurasian coots (14-109/4, 14-109/3), two Eurasian teals (15-70d/21, 15-70d/24) and fourteen swans (e.g. 15-70d/24, 14-95/6, 14-106/3) were classified as one group together with the Swedish sequence 1192 obtained from a mallard (JQ679392.1) [16], displaying 100% bootstrap support for the group now referred to as G1. The intra-genotype similarity was 100%. The proposed genotype G1 grouped closely together with genotype 1V and in relative proximity to several *C*. *abortus* isolates as well as to representatives from the established *C*. *psittaci* genotypes F and C and the provisional *C*. *psittaci* genotype YP84. The majority of the sequences including six sequences from mallards (14-105/5, 15-49d/4, 15-49d/3, 15-49d/2, 15-51d/2 14-99/6), one from a hooded crow (15-58d/2), thirty three from swans (e.g. 15-48d/9, 14-91/6, 15-64d/5) and one from a Eurasian teal (15-70d/26), clustered together with two Swedish sequences obtained from mallards (JQ679393.1, JQ679394.1) [16] with 100% bootstrap support for the group designated as G2. The position of G2 in the dendrogram resulting from the *omp*A sequence alignment is closest to *C*. *psittaci* strains of the classical ABE cluster, Matt116 and M56. G2 sequences are well separated from other genotypes as indicated by inter-genotype nucleotide distances of 0.084–0.249 compared to intra-genotype distances of only 0.001.

Finally, specimen 15-2d/1 exhibited the highest degree of identity (100%) to sequences from pigeons which were classified as *C*. *avium* (12DC97 and PV3515/3) with 100% bootstrap correspondence. The similarity to the *C*. *avium* reference strain 10DC88 from a parrot was 93.03% (results not shown).

### Isolation and molecular characterisation of representative strains of genotypes 1V, G1 and G2

In order to enable a more detailed molecular genetic analysis as prerequisite for phylogenetic reconstruction and final species assignment of genotypes 1V, G1 and G2, duplicates of *Chlamydiaceae*-positive swabs (Ct value <32) were inoculated into BGM cell culture, and chlamydiae were successfully isolated from four samples representing genotype 1V (15-58d/44), genotype G1 (15-70d/24) and genotype G2 from a swan (15-48d/9) and a mallard (15-49d/3). *Omp*A sequences were obtained from isolates and found to be 100% identical to those directly amplified from corresponding DNA samples.

**Fig 3:**
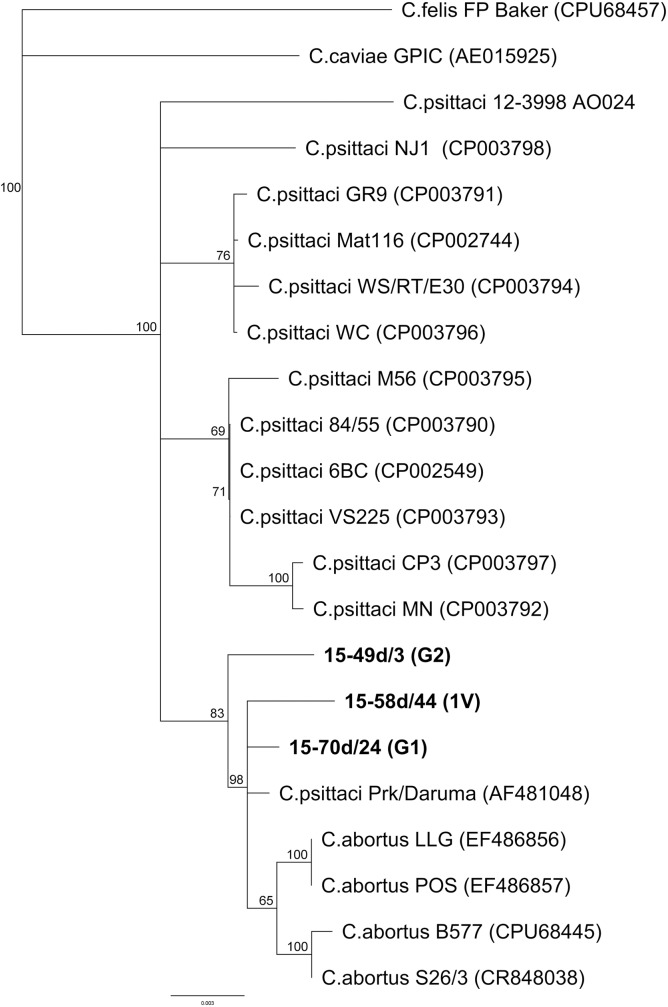
NJ dendrogram based on the global alignment of 2305 bp comprising sequences from the partial 16S rRNA gene, the intergenic spacer and the partial 23S rRNA gene. The tree was rooted on the sequence of *C*. *felis* (FP Baker). Note the close relationship between sequences of *C*. *abortus* strains from mammals and of avian genotypes G1, G2 and 1V (in bold) isolated in this study and assigned to *C*. *abortus*.

The *rrn* sequences comprising the near-complete 16S rRNA gene (1538 bp) lacking 53 bp from the 5’ end, the intergenic spacer (236–239 bp) and the first 577 bp of the 23S rRNA gene as well as MLST sequences from seven house-keeping genes were obtained from the isolated strains for further characterisation. The *rrn* sequences for 15-48d/9 and 15-49d/3 related to genotype G2 were identical. Alignment with representative sequences of *C*. *caviae*, *C*. *felis*, *C*. *psittaci* and *C*. *abortus* followed by phylogenetic tree construction clearly established the position of these isolates close to the atypical *C*.*psittaci* isolate Prk/Daruma and *C*. *abortus* strains, and well separated from the typical *C*. *psittaci* strains (**Fig 3**). This is reflected by nucleotide distance values of 0.002–0.006 and 0.004–0.009 between the three genotypes 1V, G1 and G2 and the Prk/Daruma and the *C*. *abortus* strains, respectively, in contrast to distance values of 0.007 to 0.012 between 1V, G1, G2 genotypes and typical *C*. *psittaci* strains. The nucleotide distances between the genotypes themselves ranges from 0.005 to 0.009 (see Table 2).

**Table 2:** Mean nucleotide distances in a 2305 bp fragment of the 16S-23S rRNA genes among genotypes 1V, G1, G2, atypical *C*.*psittaci* isolate Prk/Daruma, *C*. *abortus* strains, and typical *C*. *psittaci* strains.

	15-58d/44 (1V)	15-70d/24 (G1)	15-49d/3 (G2)	*C*.*psittaci* Prk/Darum.	*C*.*abortus* S26/3	*C*.*abortus* B577	*C*.*abortus* POS	*C*.*abortus* LLG	*C*.*psittaci* Mat116	*C*.*psittaci* GR9	*C*.*psittaci* WS/RT/E30	*C*.*psittaci* WC	*C*.*psittaci* NJ1	*C*.*psittaci* MN	*C*.*psittaci* CP3	*C*.*psittaci* VS225	*C*.*psittaci* 6BC	*C*.*psittaci* M56	*C*.*psittaci* 84/55
15-58d/44 (1V)																			
15-70d/24 (G1)	0.005																		
15-49d/3 (G2)	0.009	0.007																	
*C*.*psittaci* Prk/Daruma	0.004	0.002	0.006																
*C*.*abortus* S26/3	0.005	0.004	0.008	0.003															
*C*.*abortus* B577	0.006	0.005	0.009	0.004	0.001														
*C*.*abortus* POS	0.006	0.004	0.008	0.003	0.003	0.004													
*C*.*abortus* LLG	0.006	0.004	0.008	0.003	0.003	0.004	0.000												
*C*.*psittaci* Mat116	0.010	0.008	0.010	0.007	0.009	0.010	0.009	0.009											
*C*.*psittaci* GR9	0.010	0.008	0.010	0.007	0.009	0.010	0.009	0.009	0.002										
*C*.*psittaci* WS/RT/E30	0.010	0.008	0.010	0.008	0.010	0.010	0.010	0.010	0.001	0.001									
*C*.*psittaci* WC	0.010	0.007	0.010	0.007	0.009	0.010	0.009	0.009	0.001	0.001	0.002								
*C*.*psittaci* NJ1	0.012	0.010	0.011	0.010	0.011	0.012	0.011	0.011	0.006	0.006	0.006	0.005							
*C*.*psittaci* MN	0.012	0.010	0.011	0.009	0.011	0.012	0.011	0.011	0.006	0.006	0.007	0.006	0.008						
*C*.*psittaci* CP3	0.012	0.010	0.011	0.009	0.011	0.012	0.011	0.011	0.006	0.006	0.007	0.006	0.008	0.001					
*C*.*psittaci* VS225	0.010	0.008	0.010	0.008	0.010	0.010	0.010	0.010	0.004	0.004	0.004	0.003	0.006	0.005	0.005				
*C*.*psittaci* 6BC	0.009	0.007	0.008	0.007	0.008	0.009	0.008	0.008	0.003	0.003	0.003	0.002	0.005	0.003	0.003	0.001			
*C*.*psittaci* M56	0.011	0.009	0.010	0.009	0.010	0.011	0.010	0.010	0.005	0.005	0.005	0.004	0.006	0.006	0.006	0.003	0.002		
*C*.*psittaci* 84/55	0.009	0.007	0.008	0.007	0.008	0.009	0.008	0.008	0.003	0.003	0.003	0.002	0.005	0.003	0.003	0.001	0.000	0.002	

The grayscale heatmap indicates the smallest distance with black (0.000) and the largest distance (0.012) with white.

**Fig 4:**
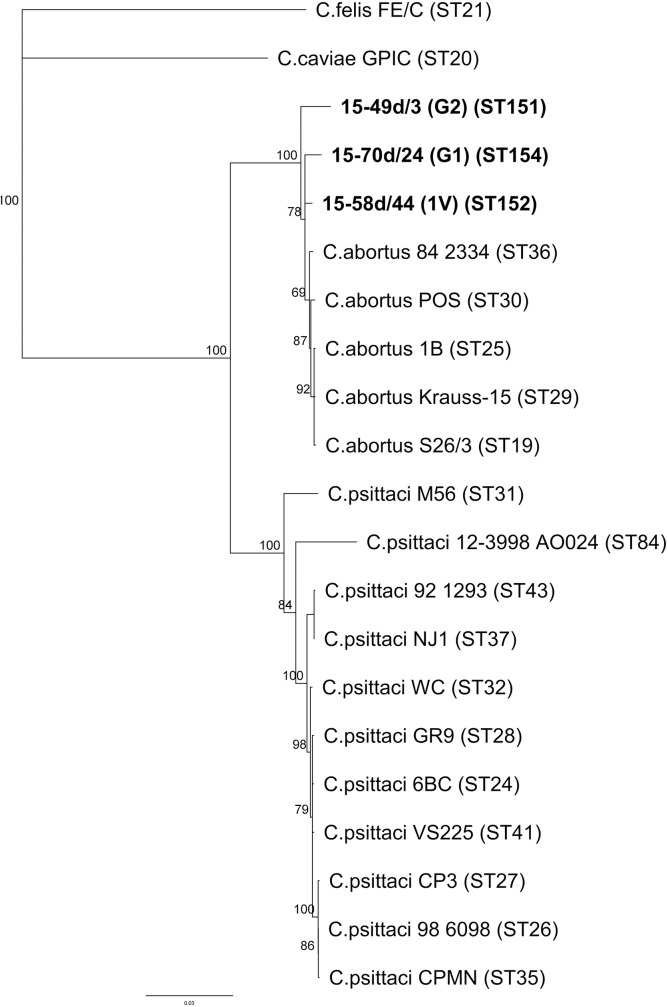
Phylogenetic tree of concatenated sequences (3104 bp) of seven MLST housekeeping gene fragments (*eno*A, *fum*C, *gat*A, *gid*A, *hem*N, *hlf*X, and *opp*A) for three isolated Chlamydia strains from this study (in bold) and established *Chlamydiaceae* species. Sequence types (ST) are given in brackets and displayed in detail for the new isolates in S2 Table. Note the separate clades for *C*. *psittaci* genotypes and *C*. *abortus* genotypes including the avian genotypes G1, G2 and 1V.

On the basis of the MLST established for *Chlamydiaceae*, four new sequence types (ST) (see **S2 Table**) have been determined from the sequences of the seven targeted genes (*eno*A, *fum*C, *gat*A, *gid*A, *hem*N, *hlf*X and *opp*A) [33] (http://pubmlst.org/chlamydiales/). MLST sequences obtained for 15-49d/3 and 15-48d/9 were identical except for a synonymous point mutation in *eno*A. Phylogenetic analysis resulted in a dendrogram (**Fig 4**) with an almost identical overall topology compared to that inferred by *rrn* sequences; 15-48d/9 and 15-49d/3 (G2), 15-70d/24 (G1) and 15-58d/44 (1V) grouped with strain 84–2334, initially described as an atypical *C*. *psittaci* strain [33], and *C*. *abortus* isolates.

## Discussion

In recent years wild animals including birds such as corvids or swans have successfully adapted to live in the specific conditions of an urban environment. Therefore, investigations into the range of prevalence of zoonotic agents like *Chlamydia* spp. are important from a public health point of view [35]. Prevalence of *Chlamydiaceae* shedders among free-living birds in Poland was 14.8% based on the results of the present study (n = 894). Previous data published by Krawiec *et al*. [21] reported a prevalence of 7.3%, however it should be noted that our survey included a higher number of samples from birds belonging to 16 families. Cloacal shedding of *Chlamydiaceae* was detected in eight families: Anatidae, Apodidae, Ciconidae, Columbidae, Corvidae, Laridae, Falconidae and Rallidae. The detection rate of *Chlamydiaceae* is in agreement with the rates reported by other researchers from Europe, although published data have shown high variability. In our study the highest number of *Chlamydiaceae* shedders was observed in the families Anatidae (19.7%) and Corvidae (13.4%). Special attention should be paid to swans belonging to the Anatidae family, as no detailed data on chlamydiosis in this species was available in the literature, while prevalence of *Chlamydiaceae* in our study was unexpectedly high with 37.1%. It can be assumed that a possible cause of the high excretion level of *Chlamydiaceae* in these birds is related to their behaviour. During winter swans dwell in the vicinity of collectors or other non-freezing water reservoirs with poor quality and feed incorrectly, e.g. on bread, which causes malnutrition and loss of fitness. A resulting weakened immunity might, in turn, lead to an increase in *Chlamydiaceae* infections. A high percentage of positive samples (33.3%) was also obtained for magpies (Corvidae family). A similar prevalence in corvids was reported by Di Francesco *et al*. [18]. Surprisingly, the percentage of positive sampled pigeons was significantly lower (4.7%) compared to previous studies conducted in other European countries. For example, about 53% of *Columba livia* tested in Madrid, Spain [36] were shedders of the pathogen. Our results are closer to data from Amsterdam in the Netherlands, where positively-sampled pigeons accounted for 7.9% [37]. *Chlamydiaceae* are shed intermittently and this may result in a high variability between values in individual European countries.

Classification of *Chlamydiaceae* positive samples in our study was initially based on specific real-time PCR. Thus, *C*. *avium* was identified in one sample from a mallard which is surprising since its occurrence had previously been observed only in pigeons and psittacine birds [14]. Further, PCR results indicated that *C*. *psittaci* was dominant in Polish wildfowl. However, more than 50% of samples were not typable by our PCR approaches which, in some cases, might be due to low DNA content, but could also be attributed to the presence of atypical chlamydial strains not covered by the available PCR assays. Indeed, *omp*A sequencing revealed that almost all chlamydial samples from Polish wild birds belong to the well-known *C*. *psittaci/C*. *abortus* group and cluster in six different clades; the typical, world-wide distributed avian *C*. *psittaci* genotypes B (pigeon, hooded crow) and C (mallard and mute swan) were present as well as the rare, provisional *C*. *psittaci* genotypes Mat116 (black-headed gull) and 1V (Eurasian magpie and hooded crow). These genotypes, so far, were only represented by single isolates [6, 10]. However, the majority of samples from this study was not assigned to any of the established genotypes, but formed two separate branches designated G1 and G2 in the *omp*A sequence-based dendrogram (**Fig 2**). Although genotyping in *Chlamydiaceae* relies upon *omp*A, the gene encoding the major protein antigen of chlamydiae, this target is unsuitable for reconstruction of phylogenetic relationships and classification, due to its polymorphic character caused by high mutation rates and frequent inter-strain recombination events [8, 38]. Instead, MLST as well as 16S rRNA, IGS and partial 23S rRNA gene analyses of representative isolates of 1V, G1 and G2 were employed to elucidate their phylogenetic position. These molecular analyses correspondingly show (**Figs 3** and **4**) the close relationship of genotypes 1V, G1 and G2 to strain 84–2334 from an Amazon parrot and genotype YP84 (represented by Prk/Daruma and further isolates from parakeets) which were previously described as intermediates between *C*. *psittaci* and *C*. *abortus* [29, 38–40]. Interestingly, the extrachromosomal plasmid inherent in most *C*. *psittaci*, but not in *C*. *abortus* strains [28], was not detected by PCR in the three representative strains of 1V, G1 and G2 (data not shown). Therefore, as previously suggested by Pannekoek and colleagues [33], we propose an expansion of the species *C*. *abortus* to include not only the classical strains isolated from mammals, but also avian isolates so far referred to as atypical *C*. *psittaci* or *C*. *psittaci/C*. *abortus* intermediates such as 84–2334, Prk/Daruma, 1V, G1 and G2. Everett and colleagues [28] in 1999 defined the species *C*. *abortus* and separated it from *C*. *psittaci* based on 16S rRNA and *omp*A gene analysis as well as host specificity, tissue tropism and pathogenicity. By this initial definition, *C*. *abortus* colonizes the placenta, causes abortion and weak neonates and is mainly hosted by sheep, goats and less frequently by cattle, pigs and horses. Our recommendation that the species *C*. *abortus* should not be defined as strictly as initially proposed is supported by the following facts:

1. Molecular analysis of a large number of avian isolates and their respective genotypes during the last 20 years indicated their close relatedness to *C*. *abortus* from mammals.
2. Taxonomic classification should reflect phylogenetic relationship and avoid the creation of paraphyletic taxa, i.e. species whose strains share their most recent common ancestor with strains of another species such as *C*. *psittaci* comprising also the intermediate genotypes according to the current definition.
3. The avian *C*. *abortus* strains seem to be of epidemiological relevance as can be deduced from their world-wide distribution in different bird families (Psittacidae, Anatidae, Corvidae, Rallidae).
4. Finally, the sister species *C*. *psittaci* is an impressive example how species definition can be expanded as it was previously described as a pathogen causing psittacosis/ornithosis in birds, but now includes also genotypes isolated exclusively from mammals (M56 and WC) as well as ruminant strains with an avian genotype causing abortions.

It has to be noted that in the newly proposed *C*. *abortus* cluster, the typical ruminant strains still constitute a monophyletic and rather homogenous group which separates clearly from the new avian members (see *rrn* nucleotide distance values in **Table 2**). In both MLST and *rrn* derived phylogenetic trees (**Figs 3** and **4**), the genotype G2 forms its own sub-branch in considerable genetic distance to ruminant *C*. *abortus* strains, whereas G1 and especially 1V share more sequence similarity with the classical strains.

The low overall genetic distance between *C*. *psittaci* and *C*. *abortus* compared to other members of the *Chlamydiaceae* family suggests that these two species have diverged much more recently from their most recent common ancestor than other chlamydial species. The detection of “intermediate” strains with a *C*. *psittaci* like phenotype regarding host range, tissue tropism and pathogenicity and a *C*. *abortus* related genotype indicates that the common ancestor of all *C*. *abortus* strains lived in birds. Most likely, a descendant was transmitted and adapted to ruminant hosts whereas others evolved further in their avian hosts. While representatives of avian *C*. *abortus* were rarely encountered in previous studies [10–11, 16, 41], the present study shows for the first time that avian genotypes of *C*. *abortus* strains are actively circulating in wild bird populations. Thus, genotype G2 was found in swans and mallards at nine independent sampling sites in Poland (**Fig 1**). Likewise, G1was detected in swans, Eurasian teals and coots at six separate locations in the country whereas genotype 1V was identified in magpies and crows at only one site. However, considering also the evidence for the presence of G1 and G2 in mallards from Sweden [16] and of 1V in crows from Russia [10], an at least Europe-wide distribution of these avian *C*. *abortus* strains in wildfowl can be assumed. Further, they seem to exhibit a considerable host specificity since genotypes G1 and G2 were predominantly detected in Anatidae and genotype 1V was exclusively found in corvids.

No observations on the health status of the sampled birds were recorded; therefore, any pathogenicity of the new avian *C*. *abortus* strains from wildfowl remains unknown. Their wide distribution, especially among swans and mallards, indicates a potential threat to domestic waterfowl via direct contact or shared water and food resources. It is, however, noteworthy, that avian *C*. *abortus* or *C*. *psittaci*/*C*. *abortus* intermediates have never been recorded in domestic waterfowl which was certainly subjected to more intensive chlamydial screenings in the past [13, 42] than wild populations. This could be ascribed to the predominance of the classical goose- and duck-specific *C*. *psittaci* genotypes C, E and E/B occupying the ecological niche of intracellular pathogens/commensals in respiratory and cloacal epithelia under the specific conditions in domestic poultry flocks. On the other hand, there is the possibility that the existence of avian *C*. *abortus* strains has just been overlooked due to insufficient diagnostic tools. Indeed, in the present study, diverse real-time PCR assays specific for *C*. *psittaci* or *C*. *abortus* [11, 26] failed to detect sequences present in the new genotypes. Sequences of genotype 1V were not amplified by any species-specific PCR, whereas sequences of genotypes G1 and G2 were amplified by the *inc*A-based *C*. *psittaci*-specific PCR with reduced sensitivity (see **S1 Table**) due to several mismatches with primers and probes. Consequently, the molecular tools available for the identification and differentiation of *C*. *psittaci* and *C*. *abortus* must be refined on the basis of new sequence alignments including the avian *C*. *abortus* strains. Because of the high sequence similarity between *C*. *psittaci* and *C*. *abortus* it is recommended to use duplex or sequential real-time PCR assays that target a sequence allowing the detection of both species first, and then amplify a sequence either specific for *C*. *abortus* or *C*. *psittaci* in a second reaction as it was proposed by Opota et al. [43].

The public health significance of the high prevalence of avian *C*. *abortus* strains in wild waterfowl can hardly be evaluated right now, because their zoonotic potential remains to be elucidated. Nevertheless, classical *C*. *psittaci* genotypes known to be readily transmitted to humans are also present in the examined wild fowl populations.

## Conclusions

*Chlamydiaceae* seem to be common in Polish wild birds, such as swans and mallards, which often live close to humans so that zoonotic implications have to be assumed. Molecular characterisation of isolates from wild birds provides a unique opportunity to understand the diversity of avian chlamydia agents. Our analyses classified the majority of chlamydiae detected in this study as new genotypes located in close phylogenetic proximity to *C*. *abortus* strains. Consequently, we propose an expansion of the *C*. *abortus* species to include not only the classical isolates of mammalian origin, but also avian isolates so far referred to as atypical *C*. *psittaci* or *C*. *psittaci/C*. *abortus* intermediates. Isolation of representatives of the new genotypes will enable further investigations into epidemiology, host preference, pathogenicity and zoonotic potential of these agents. Moreover, it will be necessary to adapt current molecular diagnostic tools to cover the detection and differentiation of *C*. *abortus* and *C*. *psittaci* strains according to the proposed taxonomy.

## Supporting information

S1 Table Identity, origin and results obtained for *Chlamydiaceae* positives samples. (XLSX)

S2 Table MLST profile of avian *C*. *abortus*. (DOCX)
