# Supplementary material for: Dissemination and genetic diversity of chlamydial agents in Polish wildfowl: Isolation and molecular characterisation of avian Chlamydia abortus strains
Source: PLoS One. 2017 Mar 28;12(3):e0174599. doi: 10.1371/journal.pone.0174599 (PMC5370153; doi:10.1371/journal.pone.0174599)
Supplement: S2 Table — (DOCX) [file pone.0174599.s002.docx]

| Isolate No | Bird species | Genotype | **ST** | Allele no. of housekeeping locus | | | | | | |
| --- | --- | --- | --- | --- | --- | --- | --- | --- | --- | --- |
|  |  |  |  | *gidA* | *enoA* | *fumC* | *gatA* | *hemN* | *hflX* | *oppA* |
| **15–49d/3** | mallard | G2 | **151** | 42 | 33 | 25 | 41 | 26 | 36 | 32 |
| **15–58d/44** | magpie | 1V | **152** | 43 | 34 | 26 | 42 | 27 | 6 | 33 |
| **15-49d/9** | mute swan | G2 | **153** | 42 | 35 | 25 | 41 | 26 | 36 | 32 |
| **15-70d/24** | Eurasian teal | G1 | **154** | 44 | 14 | 27 | 43 | 28 | 37 | 34 |

Table S2. MLST profiles of avian *C. abortus*
